# Prognosis in Pediatric Myasthenia Gravis

**DOI:** 10.15844/pedneurbriefs-34-24

**Published:** 2020-12-24

**Authors:** Jena Krueger

**Affiliations:** 1 Division of Pediatric Neurology, Helen DeVos Children’s Hospital, Grand Rapids, MI

**Keywords:** Pediatric Myasthenia Gravis, Myasthenia Antibody, Myasthenia Gravis Prognosis

## Abstract

Investigators from Oxford John Radcliff Hospital and Great Ormond Street Hospital for Children performed a retrospective study of myasthenia patients diagnosed before the age of 16 years.

Investigators from Oxford John Radcliff Hospital and Great Ormond Street Hospital for Children performed a retrospective study of myasthenia patients diagnosed before the age of 16 years. Investigators looked at demographics, clinical features, neurophysiologic testing, and antibody testing to evaluate prognostic features. The cohort included 74 patients, 69% female. Thirty-five had symptom onset by or before five years of age, and 54 had symptom onset before ten years of age. The population had a higher percentage of Afro-Caribbean, Asian, Arabic, or mixed-race backgrounds than was expected from the surrounding population. Antibodies were detected in a majority of patients (89%, 66/74). Of these patients, 52 (70%) were diagnosed via Radioimmunoprecipitation assay (RIA), and 10 (14%) were diagnosed by clustered Acetylcholine Receptors (AChR) in a cell-based assay (CBA). Three patients were found to have MuSK antibodies, and one patient had LRP4 antibodies. Eight patients (11%) were seronegative. Seronegative patients were classified by clinical symptoms and standard electrophysiologic testing, repetitive nerve stimulation, single-fiber EMG, or both. Seronegative patients also completed genetic testing to rule out common congenital myasthenic syndromes. A slight majority of the cohort presented with purely ocular symptoms. Twenty-three patients (31%) had a thymectomy performed. Seventeen (23%) of these patients reached remission.

Regarding outcome, only antibody status and repetitive nerve stimulation showed a statistically significant effect on the chance of remission. Remission was more likely in patients who were seronegative or only had antibodies identified by clustered AChR CBA than not. Remission was also more likely in patients with normal repetitive nerve stimulation at diagnosis than not. Age at onset (<10 years) and race (Asian and Caucasian) approached significance for a greater chance of remission but was not statistically significant. [1]

COMMENTARY. Juvenile myasthenia gravis is a rare disorder, and this paper represents a large cohort of pediatric patients. Overall, pediatric patients have a better prognosis than adult patients, but treatment tends to be less standardized. Seronegative patients represent a particularly challenging dilemma.

Traditional testing for acetylcholine receptor antibodies utilizes RIA, which works by radiolabeling acetylcholine receptors. The CBA increases the sensitivity of antibody testing by clustering receptors on a cell membrane, which is similar to the structure of the neuromuscular junction. The CBA can improve sensitivity for antibodies present in lower quantities or with lower binding affinity [2,3]. In one study, CBA was found to identify additional acetylcholine antibodies in 5-10% of “seronegative” patients. RIA is still recommended initially and is the only test able to quantify antibody levels [4].

The increased sensitivity of CBA could prove beneficial in younger patients. The 2016 international consensus guidelines for the management of myasthenia gravis currently recommends thymectomy be considered in children with antibody-positive myasthenia gravis [5]. CBA could prove helpful to establish the diagnosis of myasthenia gravis, distinguish between congenital and antibody-mediated syndromes and help prognosticate, even in cases presumed to be seronegative.

## Disclosures

The author has declared that no competing interests exist.
